# Supplemental Nutrition Assistance Program (SNAP)-authorized retailers received a low score using the Business Impact Assessment for Obesity and population-level nutrition (BIA-Obesity) tool

**DOI:** 10.1186/s12889-022-13624-9

**Published:** 2022-06-20

**Authors:** Bailey Houghtaling, Tessa Englund, Susan Chen, Nila Pradhananga, Vivica I. Kraak, Elena Serrano, Samantha M. Harden, George C. Davis, Sarah Misyak

**Affiliations:** 1 School of Nutrition and Food Sciences, Louisiana State University (LSU) & LSU Agricultural Center, Baton Rouge, 70803 US; 2 Thurston Arthritis Research Center, University of North Carolina at Chapel Hill, Chapel Hill, NC 27599 US; 3 Department of Nutrition, Food Science, and Packaging, San José State University, San José, CA 95192 US; 4 Department of Human Nutrition, Foods, and Exercise, Virginia Tech, Blacksburg, VA 24061 US; 5 The Virginia Cooperative Extension Family Nutrition Program, Department of Human Nutrition, Foods, and Exercise, Virginia Tech, Blacksburg, VA 24061 US; 6 Department of Agricultural and Applied Economics, Virginia Tech, Blacksburg, VA 24061 US

**Keywords:** SNAP, Public health, Corporate social responsibility, Retail food environment, Healthy food retail

## Abstract

**Background:**

The Supplemental Nutrition Assistance Program (SNAP) supports Americans with lower income to purchase dietary products at authorized retailers. This research aimed to evaluate SNAP-authorized retailers’ public commitments in support of nutrition security and to examine differences between traditional grocers and nontraditional (e.g., convenience, drug, dollar) SNAP-authorized retailers’ public commitments.

**Methods:**

Prominent United States (U.S.) SNAP-authorized retailers nationally and in two U.S. states (California and Virginia) were identified based on number of store locations (*n* = 61). Public information available in grey literature were reviewed and scored using the Business Impact Assessment for Obesity and population-level nutrition (BIA-Obesity) tool. SNAP-authorized retailers were classified as traditional (e.g., grocery) or nontraditional (e.g., non-grocery) retailers. Total BIA-Obesity from 0 to 615, representing low to optimal support) and category scores were calculated for corporate strategy, relationships with external organizations, product formulation, nutrition labeling, product and brand promotion, and product accessibility. Descriptive statistics were used to describe BIA-Obesity scores overall and by category. Mann–Whitney U was used to test for potential differences in median BIA-Obesity total scores between traditional and nontraditional SNAP-authorized retailers (a priori, *p* < 0.05).

**Results:**

Average total BIA-Obesity scores for SNAP-authorized retailers ranged from 0 to 112 (16.5 ± 23.3). Total BIA-Obesity scores for traditional SNAP-authorized retailers (32.7 ± 33.6; median 25) were higher than nontraditional SNAP-authorized retailer scores (11.2 ± 16; median 5) (*p* = 0.008). For BIA-Obesity categories, average scores were highest for the category relationships with external organizations (8.3 ± 10.3) and lowest for promotion practices (0.6 ± 2.1).

**Conclusions:**

Results of this research underscore a dearth of available evidence and substantial opportunity for improvement regarding SNAP-authorized retailer strategies to support nutrition security among Americans with lower income.

## Background

The United States (U.S.) retail food environment has many positive attributes such as efficiency in delivery, a high degree of convenience, a wide variety of choices not subject to seasonal conditions, and wide range of choices available to meet different income levels [1]. However there are also increasing concerns related to health, food security, food justice, food sovereignty, equity, and environmental and business sustainability associated with the food environment [2–5]. These issues are especially important for populations with lower income where there are observed diet-related health inequities [6–8]. As these food environment-related issues become more important [9–11], businesses are being called on to practice more corporate social responsibility (CSR) activities and incorporate these CSR activities into their business plans and decision making.

For example, a 2022 U.S. Department of Agriculture (USDA) report outlined plans to address nutrition security or the idea that “*all* Americans have consistent access to the safe, healthy, affordable foods essential to optimal health and well-being [12].” The USDA finances the Supplemental Nutrition Assistance Program (SNAP) and SNAP-Education through the Farm Bill, which provides supplemental income to households with incomes at or below 130% of the U.S. poverty threshold and mobilizes direct education and food policy, systems, and environmental changes at the local level, respectively [13, 14]. In 2019, about 35.7 million SNAP participants, of which 43% were children [15], accessed foods and beverages using SNAP benefits at over 250,000 authorized food retailers in the U.S. [16]. Given the large scope of the program, SNAP-authorized retailers are key actors to help advance USDA goals to achieve nutrition security, which moves beyond a focus on securing enough foods to emphasize nutrition and health outcomes [12, 17, 18].

Currently retailers must meet one of two eligibility criteria for SNAP authorization: 1) continuously offer a variety of staple foods in four categories including vegetables or fruits; meat, poultry, or fish; dairy products; and breads or cereals; or 2) have greater than 50% of total gross retail sales from staple food products [19]. SNAP-authorized retailers are a diverse industry regarding business model and include traditional grocery stores and nontraditional formats such as convenience, club, dollar, drug, mass merchandiser, supercenter, and other sites like certain restaurants [20–22]. Prior USDA efforts to advance nutrition security by improving the alignment of SNAP-authorized stocking standards with dietary guidelines have been contested by industry [23, 24]. This is somewhat understandable because the two eligibility requirements are rather broad and still allow for a great deal of decision making autonomy for the food retailer, and enhanced stocking standards are somewhat more restrictive. The joint product theory of CSR, as illuminated by many [25–27], effectively incorporates CSR decisions into a broader decision framework for firms that includes both CSR benefit and profit outcomes from input and output decisions. So, as in any multiproduct firm model there are then potential tradeoffs and also complementarities between CSR activities and profits (see Davis and Serrano, 2016, chapter 13 for an intermediate discussion [2]).

However, at this point we know very little about to what extent SNAP-authorized retailers even engage in CSR activities [28] and additional formative research to understand a wider array of industry practices that advance public health nutrition outcomes among both traditional and nontraditional SNAP-authorized retailers may be beneficial. Such data could inform suitable approaches for public–private partnerships, SNAP-Ed technical assistance, and/or policy strategies [9, 11, 17, 18, 28–30] to improve the SNAP-authorized food retail environment [4, 5] without compromising retailers’ ability to operate [22]. Therefore, the purpose of this investigation was to evaluate public commitments aligned with improving public health nutrition outcomes among SNAP-authorized retailers and to examine differences between traditional (grocery) and nontraditional (non-grocery) retailers.

## Methods

The present research expands upon a 2020 study that examined the availability of SNAP-authorized retailers’ commitments to use store marketing-mix and choice-architecture strategies in favor of food and beverage products aligned with the 2020–2025 Dietary Guidelines for Americans (DGA) [24, 28, 31]. This research focused on SNAP-authorized retailers in two states that were settings for a Partnership for a Healthier America campaign (California and Virginia) that aimed to improve the selection of products aligned with the DGA among consumers with lower income [32, 33]. For additional details on the selection of these sites, see Houghtaling et al., 2020 [28]. This study examined a greater variety of commitments aligned with public health nutrition goals among a greater number of SNAP-authorized retailers with considerable reach to U.S. consumers with low-income.

### Sample

The SNAP Retailor Locater database, which lists SNAP-authorized retailers’ company name and location information [34], was used to identify stores in 2017. This data was cleaned and sorted to identify the sample of SNAP-authorized retailers in California, Virginia, and at the national level. Corporate/chain SNAP-authorized retailers with the highest number of urban and rural store locations across the two states (i.e., > 4 locations) [35] and SNAP-authorized retailers with more than 300 sites nationally were selected, given their prominence in two settings for a relevant public policy campaign [32] and the U.S. food retail industry, respectively.

An iterative process during the information search (described below) was used to categorize prevalent SNAP-authorized retailers by parent corporation when needed. As an example, three of the identified companies (Food Lion, LLC, Giant, LLC, and Stop & Shop, LLC) were found to be owned by the same parent company (Koninklijke Ahold Delhaize N.V.), based on shared webpages and CSR language. This process informed how data from companies were separated or combined regarding parent corporations. Individual companies were combined with a parent company if they shared corporate language (e.g., researcher was directed to parent company webpage or report) and assessed separately if having unique corporate language that was distinct from any parent company (if applicable). This process resulted in a total of 61 unique SNAP-authorized retailers, representing the most prominent SNAP-authorized retail actors by number of store locations within California, Virginia, and nationally. Recent, publicly available sales estimates were sourced as a reference for company size and consumer reach (Table 1). Members of the research team categorized SNAP-authorized retailers by store format, including traditional grocers (*n* = 15) and nontraditional, non-grocers (*n* = 46) using criteria outlined in a 2017 USDA, Economic Research Service report on store formats and household grocery purchasing patterns [20]. Data in this USDA report suggests consumers with low income make food and beverage purchases more aligned with the DGA at grocery versus non-grocery settings [20], which may be partially influenced by the retail environment [2–5].

**Table 1:** Prominent traditional and nontraditional Supplemental Nutrition Assistance Program (SNAP)-authorized retailers with retail sales information^1^

SNAP-Authorized Retailer	Sales Estimate
**Traditional Retailers (Grocers)**	
Albertsons Companies, Inc	US$ 62.46 billion (date not available) [36]
ALDI Einkauf GmbH & Co. oHG	US$ 11.21 billion (date not available) [37]
C & K Market, Inc	US$ 300 million (date not available) [38]
H-E-B Grocery Company, LP	US$ 23.12 billion (2018) [39]
Hy-Vee, Inc	US$ 10.1 billion (2019) [40]
K-VA-T Food Stores, Inc	US$ 2.6 billion (2019) [41]
Koninklijke Ahold Delhaize N.V	US$ 66.2 billion (2019) [42]
Piggly Wiggly, LLC	US$ 750 million (date not available) [43]
Publix Super Markets, Inc	US$ 38.12 billion (2019) [44]
Save A Lot Food Stores Ltd	US$ 4 billion (date not available) [45]
Smart & Final Stores, Inc. (Smart & Final Iris Corporation)	US$ 3 billion (date not available) [46]
The Kroger Company	US$ 122.29 billion (2019) [47]
Trader Joe’s Company (Aldi Nord)	US$ 9.25 million (2019) [48]
Whole Foods Market, Inc	US$ 15.72 billion (2017) [49]
Winn-Dixie Stores, Inc	US$ 24.7 million (date not available) [50]
**Nontraditional Retailers**^2^	
7-Eleven, Inc	US$ 18.7 billion (2019) [51]
99 Cents Only Stores, LLC	US$ 5.1 million (2017) [52]
Allsup's Convenience Stores, Inc	US$ 167.6 million (date not available) [53]
Big Lots, Inc	US$ 5.2 billion (2019) [54]
Black Diamond Markets	US$ 45.4 million (2019) [55]
BP	US$ 183.5 billion (2020) [56]
Casey's General Stores, Inc	US$ 9.4 billion (2019) [57]
Chevron Corporation	US$ 146.5 billion (2019) [58]
Circle K Stores, Inc (Alimentation Couche-Tard)	US$ 59.1 million (2019) [59]
CITGO Petroleum Corporation	US$ 246 million (2019) [60]
Colonial Energy, Inc	US$ 5 million (date not available) [61]
Costco Wholesale Corporation	US$ 122.1 billion (2020) [62]
Cumberland Farms, Inc	US$ 1 billion (date not available) [61]
CVS Health	US$ 89.5 billion (2020) [63]
Dollar General Corporation	US$ 33.8 billion (2020) [64]
Dollar Tree Stores, Inc	US$ 63.2 million (2020) [65]
E&C Enterprises, Inc	US$ 161 million (date not available) [66]
Exxon Mobile Corporation	US$ 178.6 billion (2020) [67]
Family Dollar Stores, Inc	US$ 1.1 billion (2021) [68]
Fred's, Inc	US$ 1.3 billion (2019) [69]
GPM Investments, LLC	US$ 900.1 million (date not available) [69]
Kum & Go, L.C	US$ 2.6 billion (2019) [70]
Kwik Trip, Inc	US$ 5 billion (date not available) [71]
Love's Travel Stops & Country Stores, Inc	US$ 20.6 billion (2019) [70]
Marathon Petroleum Corporation	US$ 75 billion (date not available) [70]
Maverik, Inc	US$ 915.9 million (date not available) [69]
NMSO, Inc	Not available
Papa Murphy’s Holdings, Inc	US$ 126.4 million (2018) [72]
Pilot Travel Centers, LLC	US$ 29.5 billion (2019) [70]
QuikTrip Corporation	US$ 11.2 billion (2020) [70]
Racetrac Petroleum, Inc	US$ 12.6 billion (2019) [70]
Redwood Oil Company, Inc	US$ 142.3 million (date not available) [69]
Rite Aid Corp	US$ 6.2 billion (2021) [73]
Royal Dutch Shell, plc	US$ 180.5 billion (2020) [74]
Schwan's Company	US$ 3.1 billion (2017) [70]
Sears Brands	US$ 3.0 billion (2020) [75]
Sheetz, Inc	US$ 6.2 billion (2020) [70]
Speedway, LLC	US$ 33.1 billion (2019) [76]
Stewart’s Shop	US$ 310.8 million (date not available) [77]
Stripes Convenience Stores	US$ 72.2 million (date not available) [78]
Sunoco, LP	US$ 11.7 billion (date not available) [79]
Target Corporation	US$ 93.6 billion (2020) [80]
Valero Energy Corporation	US$ 27.8 billion (2021) [81]
Walgreen Company	US$ 107.7 billion (2020) [82]
Walmart, Inc	US$ 99.6 billion (2020) [83]
Wawa, Inc	US$ 13 billion (2019) [70]

^1^Sales estimates are from all sales (not only SNAP); SNAP sales by store are not public

^2^Convenience, club, dollar, drug, mass merchandiser, supercenter, and non-food stores (e.g,. restaurants) [20]

### Grey literature search

The research team searched for publicly available grey literature sources (e.g., corporate reports, newsletters, websites) to identify public commitments in alignment with public health nutrition goals from the 61 included SNAP-authorized retailers. SNAP-authorized store/company names were combined with the following search terms [28]:

“*healthy food*”; *“healthy foods”; “nutritious option”; “nutritious options”*; *“dietary choice”; “dietary choices”; “healthy choice”; “healthy choices”; fruit*; fruits; *vegetable; vegetables*; *“whole grain”; “whole grains”; “low fat dairy”*; “healthy snack”; “healthy snacks”; “healthy diet”; “healthy diets”; nutrition; health*.

The databases Access World News and LexisNexis were selected to identify press releases and results were retrieved if published during or after the year 2010, given this year marked the initiation of a Partnership for a Healthier America focus on public–private partnerships that encouraged public commitments to improve the food environment [28, 32, 33]. Searches using Google were limited to the first five pages of results based on perceived relevance of retrieved sources. Company webpages were scanned to locate information about corporate practices and commitments, including recent CSR or other reports. Searches were carried out in 2018 and again in 2020. If a SNAP-authorized retailer did not have any supporting information identified in 2018, no additional searching was completed in 2020 due to limited resources.

### Main outcome

The Business Impact Assessment for Obesity and population-level nutrition (BIA-Obesity) [30] tool was used to categorize and score SNAP-authorized retailers’ commitments that were aligned with public health nutrition outcomes. The BIA-Obesity was created by the International Network for Food and Obesity/non-communicable disease Research Monitoring and Action Support (INFORMAS) [84] and is intended to serve as a benchmarking tool to improve global nutrition accountability. Sacks et al., 2019 [30] describe the development and methodology for the BIA-Obesity, which is designed for application to diverse country contexts, and has been found sensitive to capture differences between countries [30]. The BIA-Obesity has been implemented in Australia, Canada, Malaysia, New Zealand [30, 85] France [86], and Belgium [87]. To our knowledge, the tool has not been previously used in U.S. research.

The formal process for implementing the BIA-Obesity includes: 1) selecting companies for assessment; 2) collecting publicly available information; 3) engaging with companies to identify additional information; 4) assessing companies using the BIA-Obesity tool; 5) preparing recommendations and consulting with companies; 6) providing results to companies privately; and 7) a public release of findings. In this study, steps 3, 5, and 6 were not conducted due to time and resource constraints. Given social corporate responsibility commitments to help improve population nutrition often result in promotional materials for retailers [32] and evidence showing consumers’ growing interest in health [88], it was expected that most information pertaining to the BIA-Obesity would be publicly available.

The BIA-Obesity scores industry commitments regarding public health nutrition outcomes across six categories: corporate strategy; relationships with external organizations; product formulation; nutrition labeling; product and brand promotion; and product accessibility [30]. Brief definitions follow.

Corporate strategy: policies and commitments related to: “addressing obesity and improving population-level nutrition [30],” such as in company mission statements;

Relationships with external organizations: “support provided to external groups (e.g., professional associations, research organizations, community, and industry groups) related to health and nutrition [30]”;

Product reformulation: “product development and reformulation to reduce nutrients of concern (i.e., sodium, free sugars, saturated fat, trans fat) and energy content [30],” regarding any store brand products;

Nutrition labeling: “the disclosure and presentation of nutrition information on product packaging, online, and on menus,” regarding online information about any quick-service foods and beverages that may be sold in stores, for example [30];

Product and brand promotion: “reducing the exposure of children (aged <18) and adults to promotion of “less healthy” foods/brands [30]”;

Product accessibility: “the availability and affordability of healthy compared with “less healthy” foods” [30].

The standardized BIA-Obesity scoring system captures the availability and strength of supporting language across these categories. Scores for each of the individual categories are summed to calculate the total BIA-Obesity score, with a possible range of zero (no support for public health nutrition outcomes) to 615 (optimal support).

The research team used an internal system to prioritize, assess, and score the gathered evidence, given the scope of SNAP-authorized retailers’ information was not always clear. Evidence sources were prioritized as follows: company commitments in published reports were assumed to be nationally-reaching and were prioritized for BIA-Obesity data; webpage information about company commitments were extracted to the BIA-Obesity if the information was not detailed in reports; and press release information were extracted to the BIA-Obesity if this language was not captured in reports or on webpages, given this information may have been specific to local settings or for a limited time. Given the assumption that information about commitments found on webpages and/or press releases, but not in reports, were limited in reach, these sources at times resulted in lower scores following the BIA-Obesity criteria [30]. This process was conducted among a team of four researchers. Data was independently extracted to the BIA-Obesity and scored among two researchers, with agreement reached through discussion. A fifth researcher helped to settle discrepancies.

### Data analysis

SPSS version 25 was used for data analysis (IBM Corporation, Armonk, NY). Means and standard deviations were calculated for total BIA-Obesity scores and corporate strategy, relationships with external organizations, product formulation, nutrition labeling, product and brand promotion, and product accessibility category subscores. A non-normal distribution for scores among traditional (grocers) (*n* = 15) and nontraditional (non-grocers) (*n* = 46) formats was indicated using the Kolmogorov–Smirnov test, which was chosen based on group sample size (< 50) [89]. Therefore, Mann–Whitney U was used to test for a potential difference in median total scores (continuous variable) by store format (traditional or nontraditional) [90]. Significance was set a priori at *p* < 0.05.

## Results

The BIA-Obesity scores by subcategory and total scores for 61 SNAP-authorized retailers are shown in Tables 2. Total BIA-Obesity scores ranged from 0 to 112, with an average score of 16.5 (Table 3). The relationships with external organizations category received the highest score, on average, compared to all other BIA-Obesity categories (mean 8.3 out of a possible score of 40). The product and brand promotion category received the lowest score on average (mean 0.6 out of a possible score of 12.5) (Table 3). Differences were found in total BIA-Obesity scores by SNAP-authorized format (*p* = 0.008). Traditional (i.e., grocers) SNAP-authorized retailers scored higher (32.7 ± 33.6; median 25) than nontraditional (i.e., non-grocers) SNAP-authorized retailers (11.2 ± 16; median 5).

**Table 2:** Prominent Supplemental Nutrition Assistance Program (SNAP)-authorized retailers’ scores using the Business Impact Assessment—Obesity and population-level nutrition (BIA-Obesity) tool (*n* = 61)

Corporate/Chain SNAP-Authorized Retailer	**BIA-Obesity Categories (Top Possible Score)**						Total Score (615)
	Corporate Strategy (30)	Relationships with External Organizations (80)	Product Formulation (85)	Nutrition Labeling (145)	Product and Brand Promotion (155)	Product Accessibility (120)	
**Traditional Retailers (Grocers)**							
Albertsons Companies, Inc	4	40	10	25	0	7.5	86.5
ALDI Einkauf GmbH & Co. oHG	12.5	25	10	19	7.5	0	74
C & K Market, Inc	0	0	0	0	0	0	0
H-E-B Grocery Company, LP	5	20	0	7.5	2.5	5	40
Hy-Vee, Inc	2.5	15	0	7.5	0	0	25
K-VA-T Food Stores, Inc	0	10	0	3.5	2.5	0	16
Koninklijke Ahold Delhaize N.V	12	40	5	40	12.5	2.5	112
Piggly Wiggly, LLC	0	0	0	0	0	0	0
Publix Super Markets, Inc	0	25	0	10	0	0	35
Save A Lot Food Stores Ltd	0	10	0	0	0	2.5	12.5
Smart & Final Stores, Inc	0	0	0	0	0	0	0
The Kroger Co	7.5	10	0	5	0	0	22.5
Trader Joe’s	0	15	2.5	5	5	0	27.5
Whole Foods Market, Inc	0	20	5	5	5	0	35
Winn-Dixie Stores, Inc	0	5	0	0	0	0	5
**Nontraditional Retailers**^1^							
7-Eleven, Inc	0	0	0	0	0	0	0
99 Cents Only Stores, LLC	0	5	0	0	0	0	5
Allsup's Convenience Stores, Inc	0	0	0	0	0	0	0
Big Lots, Inc	0	0	0	0	0	0	0
Black Diamond Markets	0	0	0	0	0	0	0
BP, plc	0	0	0	0	0	0	0
Casey's General Stores, Inc	0	0	0	0	0	0	0
Chevron Corporation	0	5	0	0	0	0	5
Circle K Stores and Alimentation Couche-Tard	0	20	0	2.5	0	0	22.5
CITGO Petroleum Corporation	0	15	0	0	0	0	15
Colonial Energy, Inc	0	20	0	0	0	0	20
Costco Wholesale Corporation	0	5	0	10	0	0	15
Cumberland Farms, Inc	0	0	0	7.5	0	5	12.5
CVS Health	7.5	25	10	7.5	0	12.5	62.5
Dollar General Corporation	0	0	0	0	0	0	0
Dollar Tree Stores, Inc	0	10	0	0	0	0	10
E&C Enterprises, Inc	0	0	0	0	0	0	0
Exxon Mobile Corporation	0	5	0	0	0	0	5
Family Dollar Stores, Inc	0	0	0	0	0	0	0
Fred's, Inc	0	0	0	0	0	0	0
GPM Investments, LLC	0	0	0	10	0	0	10
Kum & Go, L.C	0	5	0	10	0	0	15
Kwik Trip, Inc	0	0	0	2.5	0	5	7.5
Love's Travel Stops & Country Stores, Inc	0	0	0	0	0	0	0
Marathon Petroleum Corporation	0	10	0	0	0	0	10
Maverik, Inc	0	0	0	0	0	0	0
NMSO, Inc	0	0	0	0	0	0	0
Papa Murphy’s Holdings, Inc	0	0	0	5	0	0	5
Pilot Travel Centers, LLC	0	20	0	10	0	0	30
QuikTrip Corporation	0	0	0	5	0	0	5
Racetrac Petroleum, Inc	0	0	0	0	0	0	0
Redwood Oil Company, Inc	0	0	0	2.5	0	0	2.5
Rite Aid Corp	0	20	0	10	0	0	30
Royal Dutch Shell, plc	0	0	0	0	0	0	0
Schwan's Company	0	5	0	5	0	0	10
Sears, Roebuck and Co	0	10	0	0	0	0	10
Sheetz, Inc	7.5	10	0	22.5	2.5	7.5	50
Speedway, LLC	0	0	0	7.5	0	0	7.5
Stewart’s Shops	0	5	0	0	0	0	5
Stripes Convenience Stores	0	0	0	5	0	0	5
Sunoco, LP	0	0	0	0	0	0	0
Target Corporation	0	10	0	0	0	7.5	17.5
Valero Energy Corporation	0	10	0	0	0	0	10
Walgreen Company	0	25	0	13.5	0	0	38.5
Walmart, Inc	7.5	25	30	5	0	0	67.5
Wawa, Inc	0	5	0	0	0	0	5

^1^Convenience, club, dollar, drug, mass merchandiser, supercenter, and non-food stores (e.g., restaurants) [20]

**Table 3:** Descriptive values for Business Impact assessment for Obesity and population-level nutrition (BIA-Obesity) scores among 61 prominent Supplemental Nutrition Assistance Program (SNAP)-authorized retailers in the United States

Category	Minimum Score	Maximum Score	Mean Score	Standard Deviation
Corporate Strategy	0	12.5	1.1	2.9
Relationships	0	40	8.3	10.3
Product Formulation	0	30	1.2	4.4
Nutrition Labeling	0	40	4.4	7.3
Product and Brand Promotion	0	12.5	0.6	2.1
Product Accessibility	0	12.5	0.9	2.5
**Total Score**	0	112	16.5	23.3

## Discussion

Prominent SNAP-authorized retailers in two U.S. states and nationally were included in this research, which aimed to assess company commitments aligned with public health nutrition outcomes using a standard BIA-Obesity tool. Understanding if BIA-Obesity scores differed between traditional and nontraditional SNAP-authorized retailers was also a priority, given the diverse business models of SNAP-authorized retailers in the United States [20, 34], differences in the nutritional quality of consumer purchases by store format [20], and the need for targeted efforts to improve industry commitments to help achieve nutrition security [12, 22].

Overall, the BIA-Obesity scores resulting from this research suggest SNAP-authorized retailers have not maximized opportunities to improve nutrition security among households with low income in the U.S., despite an increased emphasis on public–private strategies to improve the food environment since 2010 [12, 28, 32, 33]. Prior research that examined SNAP-authorized retailers’ commitments to use marketing-mix and choice-architecture strategies to encourage consumers’ selection of foods and beverages aligned with the DGA also found limited language in support of these efforts [28]. Given the importance of the SNAP-authorized retail sector in helping to achieve nutrition security and the Sustainable Development Goals [9, 11, 12, 85], it is important for future work to build off BIA-Obesity results to understand what potential solutions may improve food retail environments while advancing (or at least not impeding) private- and public-sector interests.

Researchers in Canada also found low scores when using the BIA-Obesity to assess food and beverage manufacturer practices (ranged from 4 to 60% of the top possible score) [91]. In the present research, traditional (grocery) SNAP-authorized retailers’ public commitments were found to have more language in support of public health nutrition goals, resulting in higher BIA-Obesity scores relative to nontraditional (non-grocery) SNAP-authorized retailers. However, the highest total score was only 18% of the total possible BIA-Obesity score. As populations with lower incomes have been found to rely on nontraditional stores for household food and beverage purchases more than populations with higher incomes [20, 92], these settings/sectors should be key points for intervention based on the limited, and often no public language, in support of public health nutrition outcomes.

In the context of the joint CSR and Profit framework, what strategies are actually effective will be heterogeneous and depend on the market and the strategy. In the best case scenario of the “strategic case” [25] CSR activities will improve profits and thus reinforce each other. Alternatively, even in the non-strategic cases, where there can be tension between CSR activities and profitability, CSR strategies can still affect business decisions if the firm places sufficient weight on these activities or the ‘dosage’ of these activities are sufficient. There are numerous activities that could be considered. For example, strategies to improve retailers’ commitments in support of public health nutrition efforts may include voluntary public–private partnership efforts, such as through the Partnership for a Healthier America, which could use the BIA-Obesity to guide retail partnership agreements [33]. SNAP-Ed technical assistance, which will be better financed as a result of USDA’s nutrition security efforts [12], may also be a worthwhile approach to improve company commitments regarding public health nutrition, especially among regional or local chains [21]. Last, given the relatively short time-frame to meet enormous societal goals for food system transformation [8, 10], use of regulatory strategies by the USDA may also prove appropriate to incentivize or disincentivize SNAP-authorized retailers’ corporate practices to support nutrition security and public health. While it is outside the scope of this article to suggest specific solutions and related accountability strategies, both the BIA-Obesity and the marketing-mix and choice-architecture framework used in prior research [28, 31] should be used to evaluate and track the success of strategies to improve SNAP food environments and ultimately nutrition security. This preliminary work can serve as a baseline to assess future changes.

### Limitations

There are several limitations of this work. BIA-Obesity process steps 3 (engaging with companies to identify additional information), 5 (preparing recommendations and consulting with companies), and 6 (providing results to companies privately) were not implemented due to resource constraints. These steps could be completed using qualitative inquiry to provide additional context to the identified gaps found in the present research. Future work could also seek to validate the BIA-Obesity for a U.S. context, as this was beyond the scope of the present work given limited evidence identified to inform changes. However, certain BIA-Obesity indicators, such as commitments around reducing trans fat in products were unlikely to show up in SNAP-authorized retailers’ public commitments due to U.S. policies to eliminate artificial trans fat from the food system [93]. This likely led to lower BIA-Obesity scores being recorded among all retailers. Last, analysis regarding traditional versus nontraditional formats could be more nuanced [94]. However, certain nontraditional formats were not frequently observed in this research (e.g., only one prominent SNAP-authorized retailer was classified as a club format), which limited opportunities to understand variations among several nontraditional formats. Despite these limitations, this work used a robust search strategy to apply the BIA-Obesity to a U.S. and SNAP context for the first time and can be used as a baseline measure to help assess and inform future efforts to improve nutrition security.

## Conclusion

Results of this research underscore a dearth of available evidence and substantial opportunity for improvement regarding SNAP-authorized retailer strategies to support nutrition security among Americans with lower income.

## Data Availability

The SNAP-authorized retail data used to identify prominent SNAP-authorized retailers in this research was accessed online: https://snaped.fns.usda.gov/library/materials/snap-retailer-locator. Public access to this database is open.

## Abbreviations

U.S.: United States
CSR: Corporate Social Responsibility
USDA: United States Department of Agriculture
SNAP: Supplemental Nutrition Assistance Program
DGA: Dietary Guidelines for Americans
BIA-Obesity: Business Impact Assessment for Obesity and population-level nutrition
INFORMAS: The International Network for Food and Obesity/non-communicable disease Research Monitoring and Action Support
